# Mouse mast cell protease 4 suppresses scar formation after traumatic spinal cord injury

**DOI:** 10.1038/s41598-019-39551-1

**Published:** 2019-03-06

**Authors:** Tim Vangansewinkel, Stefanie Lemmens, Nathalie Geurts, Kirsten Quanten, Dearbhaile Dooley, Gunnar Pejler, Sven Hendrix

**Affiliations:** 1 Department of Morphology, Biomedical Research Institute, Hasselt University, Diepenbeek, Belgium; 2 Health Science Centre, School of Medicine, University College Dublin, Dublin, Ireland; 3 Department of Anatomy, Physiology and Biochemistry, Swedish University of Agricultural Sciences, Uppsala, Sweden; 4 Department of Medical Biochemistry and Microbiology, Uppsala University, Uppsala, Sweden

## Abstract

Spinal cord injury (SCI) triggers the formation of a glial and fibrotic scar, which creates a major barrier for neuroregenerative processes. Previous findings indicate that mast cells (MCs) protect the spinal cord after mechanical damage by suppressing detrimental inflammatory processes via mouse mast cell protease 4 (mMCP4), a MC-specific chymase. In addition to these immunomodulatory properties, mMCP4 also plays an important role in tissue remodeling and extracellular matrix degradation. Therefore, we have investigated the effects of mMCP4 on the scarring response after SCI. We demonstrate that the decrease in locomotor performance in mMCP4^−/−^ mice is correlated with excessive scar formation at the lesion. The expression of axon-growth inhibitory chondroitin sulfate proteoglycans was dramatically increased in the perilesional area in mMCP4^−/−^ mice compared to wild type mice. Moreover, the fibronectin-, laminin-, and collagen IV-positive scar was significantly enlarged in mMCP4^−/−^ mice at the lesion center. A degradation assay revealed that mMCP4 directly cleaves collagen IV *in vitro*. On the gene expression level, neurocan and GFAP were significantly higher in the mMCP4^−/−^ group at day 2 and day 28 after injury respectively. In contrast, the expression of fibronectin and collagen IV was reduced in mMCP4^−/−^ mice compared to WT mice at day 7 after SCI. In conclusion, our data show that mMCP4 modulates scar development after SCI by altering the gene and protein expression patterns of key scar factors *in vivo*. Therefore, we suggest a new mechanism via which endogenous mMCP4 can improve recovery after SCI.

## Introduction

Spinal cord injury (SCI) is a chronic disorder that not only results in functional impairments and loss of sensation below the lesion site, but can also cause neuropathic pain and incontinence^1,2^. Currently, there are no effective therapies capable of restoring lost functions after injury. A major impediment for regenerative processes after SCI can be attributed to the expression of inhibitory factors that are associated with the lesion scar^3,4^. The scarring response is an evolving process, which involves various cells that accumulate at the lesion site (*e.g*. astrocytes, oligodendrocyte precursors, pericytes, ependymal cells) at different time points after injury^5–10^. These cellular responses result in the local deposition of extracellular matrix (ECM) components that form a dense scar. This scar is characterized by the expression of axon-growth inhibitory chondroitin sulfate proteoglycans (CSPGs) in the perilesional area^11,12^ and the formation of a collagenous and basement membrane-rich matrix in the lesion center, contributes to a physical barrier^6,13,14^. Although scar tissue displays favorable effects in the acute phase after SCI by restoring the tissue integrity and limiting secondary tissue damage^6,15^, it also blocks axon regeneration and other regenerative processes at later stages. Scar remodeling therapies are therefore of great interest in the SCI research field.

Mast cells (MCs) are immune cells characterized by electron-dense granules in their cytoplasm within which preformed mediators are stored, including cytokines and several MC-specific proteases (*i.e*. chymase and tryptase)^16–20^. MCs reside in virtually all organs, including the brain and spinal cord^21,22^. As effector cells of the innate immune system, MCs from the periphery can also infiltrate the central nervous system (CNS) through a compromised blood brain barrier (BBB) which is characteristic of many neuroinflammatory diseases and traumatic injuries^23–25^. As reviewed in Nelissen *et al*.^26^, MCs and their secreted mediators can modulate the inflammatory processes in multiple CNS pathologies. Amongst their complex effects, they can either contribute to neurological damage or provide neuroprotection. We have previously provided strong evidence demonstrating that MCs exert beneficial effects after traumatic CNS injury. Experiments in knockout mice indicated that MCs support neuronal survival and functional recovery after traumatic CNS injuries^27,28^. In particular, the protective effects of MCs appeared to be attributed due to their ability to promote the degradation of inflammation-associated cytokines such as interleukin 6 (IL-6) and monocyte chemoattractant protein-1 (MCP-1), thereby tempering ‘detrimental’ inflammatory processes. These immunomodulatory effects were partly mediated via mouse mast cell protease 4 (mMCP4)^28^. This protease is the murine homolog of human α-chymase^29^; and it is a serine protease with chymotrypsin-like cleavage specificity^30,31^. A similar role for mMCP4 in the early inflammatory phase of experimental autoimmune encephalomyelitis (a mouse model of multiple sclerosis) has been reported, indicating immunomodulatory capacities also in other neurodegenerative diseases^32^.

Apart from its effects on the inflammatory response, mMCP4 is involved in ECM remodeling through direct cleavage of ECM components or, indirectly, by activating other ECM-processing enzymes (e.g. matrix metalloproteinases)^16,33^. By taking the ECM-degrading properties of mMCP4 into consideration, in this study, we investigated whether mMCP4 improves recovery after SCI by targeting the inhibitory lesion scar. We demonstrate that the absence of mMCP4 results in exacerbated scarring levels at the lesion site, suggesting an additional modulation effect of mMCP4. Hence, these data introduce a new potential mechanism via which MC chymase can alter the scar environment and support functional recovery after traumatic SCI.

## Results

### Impaired locomotor performance and increased scar formation in mMCP4 knockout mice after SCI

**Figure 1:**
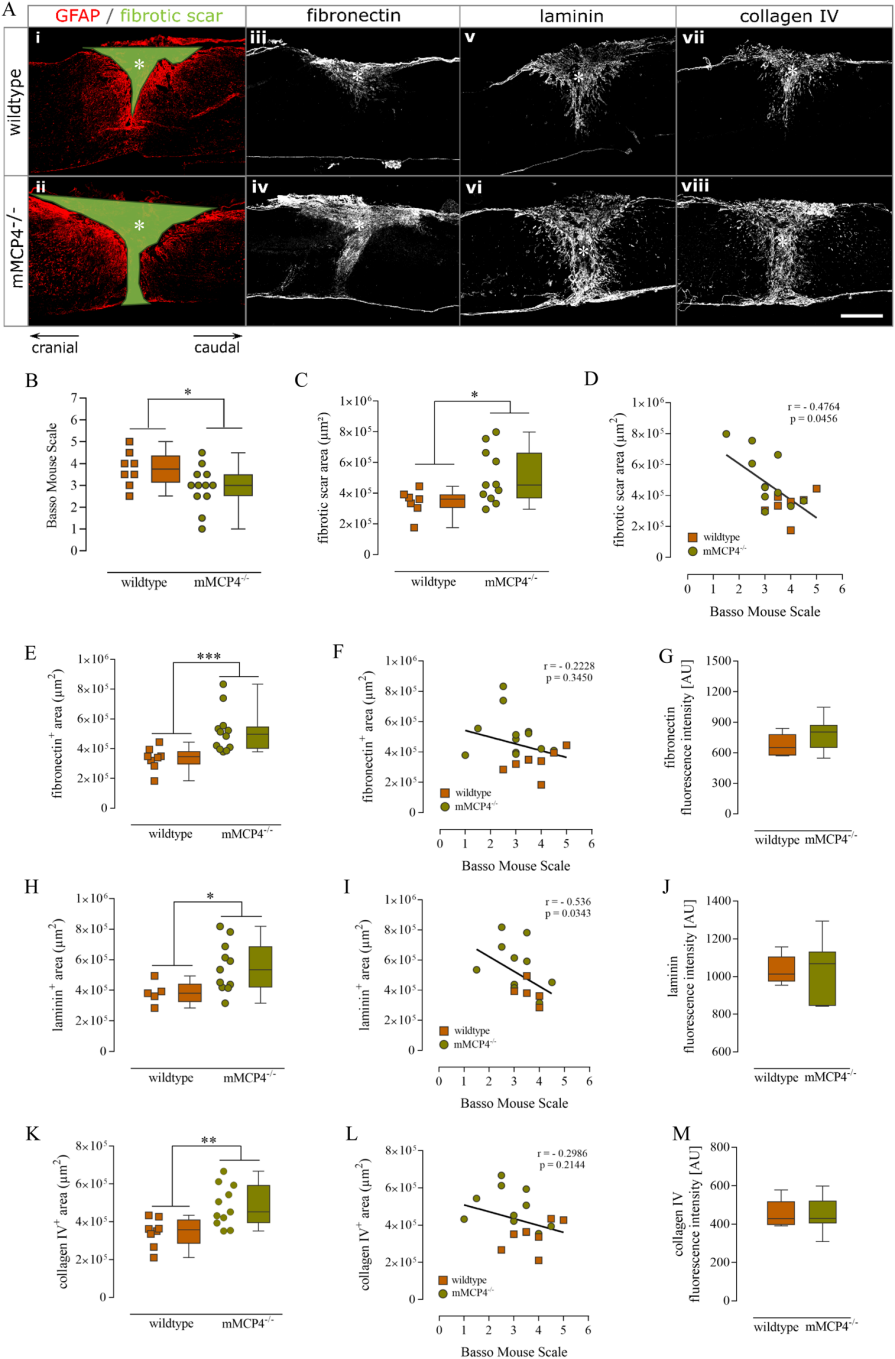
Impaired functional recovery and increased fibrotic scar formation in mMCP4 knockout mice after SCI. (**A**) Representative fluorescent photomicrographs of the fibrotic scar at the lesion site in WT and mMCP4^−/−^ mice, respectively. Scale bar in all images = 500 µm. GFAP is visualized in red in Ai/ii. **(B**) The deficiency of mMCP4 in mMCP4 knockout mice results in a significantly decreased BMS score after SCI. WT mice: n = 8; mMCP4^−/−^ mice: n = 12. (**C**) A significant increase in fibrotic scar area (i.e. GFAP negative area marked in green in Ai/ii) was observed in mMCP4 knockout mice (Aii) compared to WT controls (Ai) at 28 dpi. **(D)** Inverse correlation between fibrotic scar formation and functional outcome after SCI in our mouse model (p = 0.0456, r = −0.4764, Spearman rank correlation coefficient). **(E**,**H**,**K**) To characterize the fibrotic scar response in more detail, we examined the expression of fibronectin and of the basement membrane components laminin and collagen IV. We found that the fibronectin- (**E**), laminin- (**H**) and collagen IV-positive (**K**) area were significantly increased in mMCP4^−/−^ mice (Aiv/vi/viii) compared to WT mice (Aiii/v/vii). **(F**,**I**,**L**) A significant inverse correlation was observed between the laminin-positive area and the functional outcome (p = 0.0343, r = −0.536) (**I**), but not with the other matrix components (**F**,**L**). **(G**,**J**,**M**) In contrast to the area, the intensity of the immunoreactivity of these extracellular matrix components at the lesion was comparable between WT and mMCP4^−/−^ mice. Individual data points are shown per mouse, together with the corresponding boxplots with the median and whiskers indicating the minimum and maximum. Histological analyses were performed on 5–8 WT mice and 10-12 mMCP4^−/−^ mice. AU: arbitrary units. Asterisks in the fluorescent images indicate the lesion center. *p < 0.05; **p < 0.01; ***p < 0.001.

**Figure 2:**
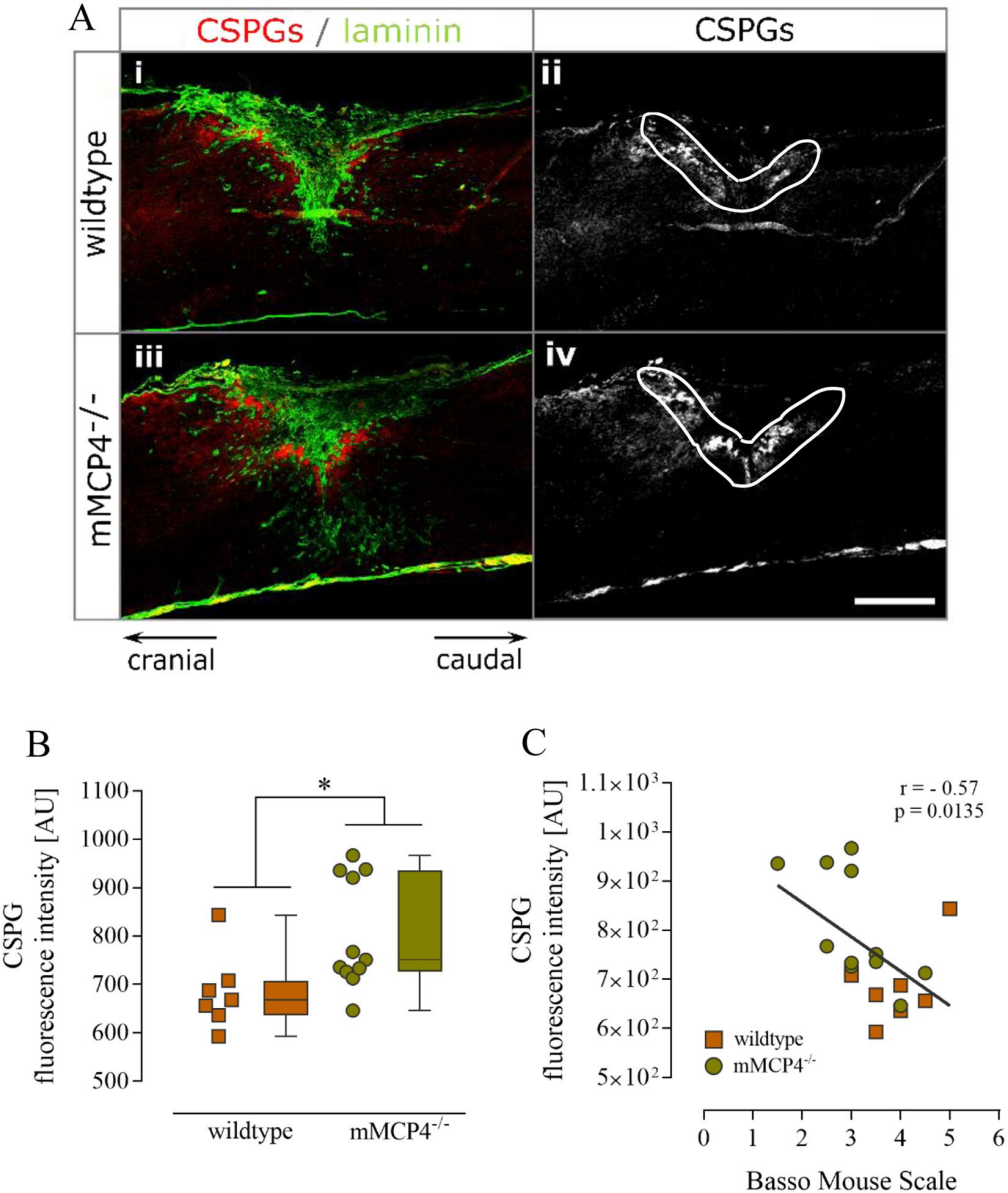
Increased expression of chondroitin sulfate proteoglycans in mMCP4 knockout mice after SCI. **(A)** Representative fluorescent photomicrographs of CSPG expression at the lesion site in WT and mMCP4^−/−^ mice, respectively. Scale bars = 500 µm. Immunofluorescence for laminin (green) was performed to highlight the lesion center, and CSPGs (red) are strongly upregulated in the perilesional area after traumatic SCI (Ai/iii). (**B**) A significant increase in CSPG immunoreactivity was observed in the perilesional area in mMCP4^−/−^ (Aiii/iv) compared to WT mice (Ai/ii) (area encircled by white line in Aii/iv indicates the analyzed region). **(C**) Inverse correlation between CSPG expression at the lesion site and the functional outcome after SCI in our mouse model (p = 0.0135, r = −0.57, Spearman rank correlation coefficient). Individual data points are shown per mouse, together with the corresponding boxplots with the median and whiskers indicating the minimum and maximum. WT mice: n = 7; mMCP4^−/−^ mice: n = 11. AU: arbitrary units. Asterisks in the images indicate the lesion center. *p < 0.05.

To determine the effect of mMCP4 on scar formation after SCI, we performed an *in vivo* experiment in which mMCP4^−/−^ mice and their corresponding wild type (WT) controls were subjected to a dorsal T-cut hemisection lesion as described in the Methods section. A significant decrease in hind limb locomotor functions was observed in mMCP4 knockout mice compared to WT mice at 28 days post injury (dpi) (Fig. 1B). Histological analysis revealed that the fibrotic scar area (i.e. the GFAP negative area marked in green in Fig. 1Ai,ii) was significantly increased in mMCP4^−/−^ mice compared to WT control mice (Fig. 1Ai/ii,C). We also observed an inverse correlation between fibrotic scar formation and the functional outcome after SCI in our mouse model (p = 0.0456, r = −0.4764, Spearman rank correlation coefficient) (Fig. 1D). To analyze the fibrotic scar in more detail, we measured the area and immunoreactivity of key scar components at the lesion site, namely fibronectin, laminin and collagen type IV. We found that the fibronectin- (Fig. 1Aiii/iv,E), laminin- (Fig. 1Av/vi,H) and collagen IV-positive areas (Fig. 1Avii/viii,K) were significantly increased in mMCP4^−/−^ compared to WT mice at 28 dpi. Moreover, also a significant inverse correlation was observed between the laminin-positive area and the functional outcome (p = 0.0343, r = −0.536) (Fig. 1I), but not with the other matrix components Fig. 1F,L). The intensity of staining for these scar components (within the positive area) has been analyzed as well and was comparable between mMCP4^−/−^ and WT mice (Fig. 1A,G,J,M). Lastly, we analyzed the expression of axon-growth inhibitory CSPGs in a specifically-defined area around the lesion center (white encircled area in Fig. 2Aii/iv). Immunoreactivity for CSPGs was significantly higher in the perilesional area in mMCP4^−/−^ mice compared to WT mice at 28 dpi (Fig. 2Aii/iv,B). Moreover, we also found a correlation between an increase of CSPG expression in the perilesional area and functional impairment after SCI, indicating an important role of CSPGs in SCI pathology (p = 0.0135, r = −0.57) (Fig. 2C).

### Selected scar-associated ECM components are cleaved by mMCP4 *in vitro*

**Figure 3:**
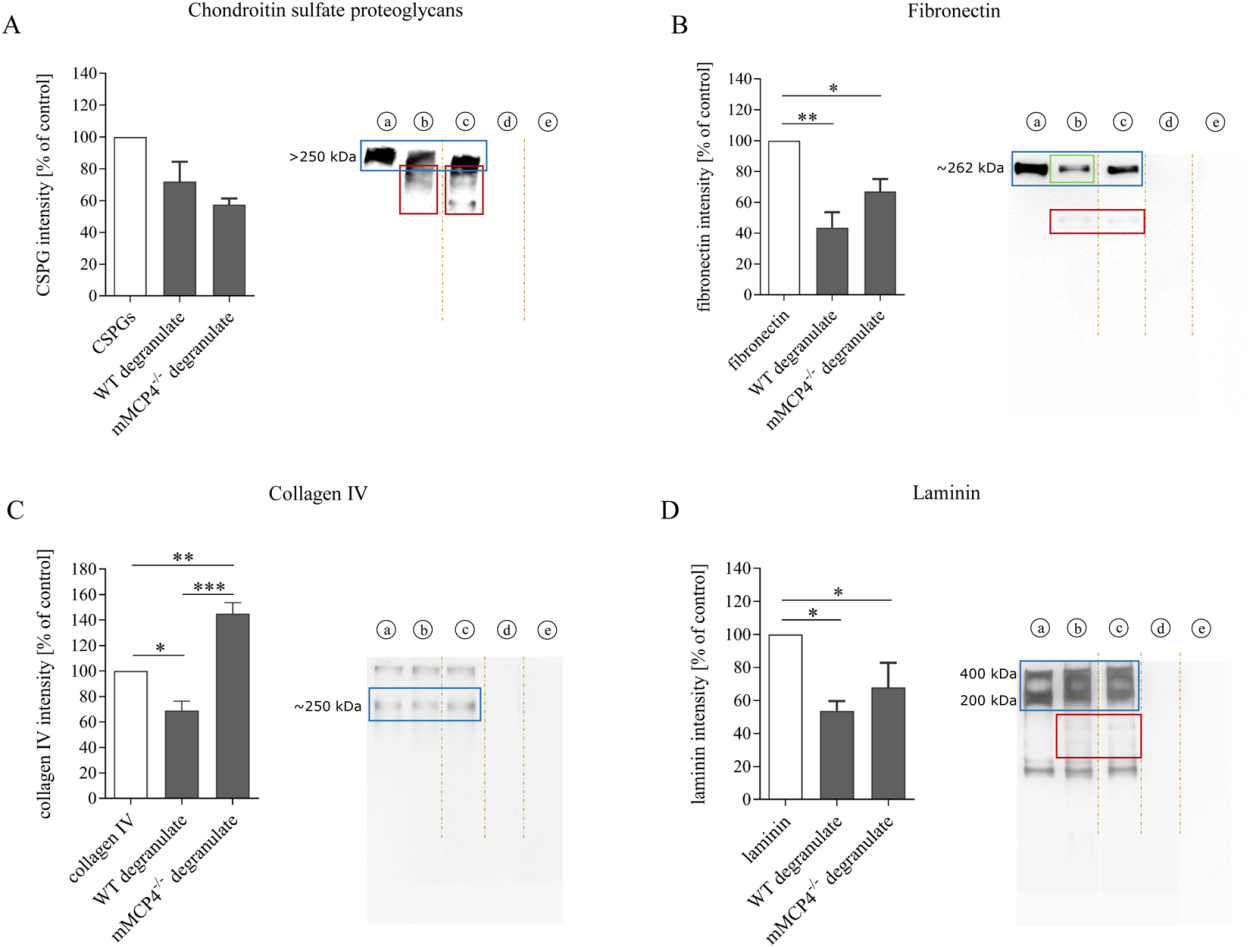
mMCP4 degrades scar-associated ECM components *in vitro*. **(A**–**D)** Recombinant fibronectin, collagen IV, laminin or a CSPG-mix (aggrecan, neurocan, phosphacan, versican) were incubated with degranulate from WT or mMCP4^−/−^ MCs to measure protein degradation. **(A)** Fragments of CSPG degradation products are visible at lower molecular weight levels after incubation with degranulate from WT or mMCP4^−/−^ MCs (red-boxed areas in A). No statistically significant difference in CSPG intensity was observed between the groups. **(B)** Fibronectin was cleaved by degranulate from both WT and mMCP4^−/−^ MCs (the red-boxed area indicates cleavage fragments); although with reduced cleavage (trend) by degranulate from mMCP4^−/−^ vs. WT MCs (the green box indicates a stronger reduction in intensity of the fibronectin protein band after incubation with MC_WT_ degranulate). **(C)** Collagen IV was cleaved by degranulate from WT MCs, but not degranulate from mMCP4^−/−^ MCs. **(D)** Laminin was cleaved by degranulate from both WT and mMCP4^−/−^ MCs (the red box indicates cleavage fragments). Data were normalized to the control condition (a) and presented as mean ± SEM; n = 3-4 experimental repeats/condition; *p < 0.05, **p < 0.01, ***p < 0.001. Legend: (a) = recombinant protein; (b) = recombinant protein + MC_WT_ degranulate; (c) = recombinant protein + MC_mMCP4_^−/−^ degranulate; (d) = degranulate from WT MCs alone; (e) degranulate from mMCP4^−/−^ MCs alone. Intensity analysis were performed on the main protein bands that correspond with the known molecular weights of the ECM components (blue boxes). Brown dotted lines in the images indicate that the original blots have been cropped to exclude data on mMCP6 degradation which are not the focus of this study and have been published in Vangansewinkel *et al*.^34^. Original uncropped Western blot images are shown in Supplementary Fig. S1.

An *in vitro* degradation assay was performed to determine which scar-associated ECM components present as substrates for mMCP4. Recombinant fibronectin, laminin, collagen IV or a mix of CSPGs (aggrecan, neurocan, phosphacan, versican) were incubated with MC degranulate collected from either WT mice or mMCP4^−/−^ mice. Cleavage fragments were visible after incubation with degranulate from both WT and mMCP4^−/−^ MCs (red-boxed areas in Fig. 3A), although quantification did not reveal any statistically significant effect of mMCP4-deficiency on the extent of CSPG degradation. This indicates that mMCP4 does not directly cleave CSPGs in our *in vitro* model. The protein band of fibronectin (262 kDa) shows a decrease in intensity after incubation with degranulate from WT MCs (green-boxed area, Fig. 3B), and also cleavage products were observed at lower molecular weight levels (red-boxed area, Fig. 3B). When incubated with degranulate from mMCP4^−/−^ MCs, considerably less degradation of fibronectin was visible (Fig. 3B). However, the differences in degradation between degranulate from WT MCs and mMCP4^−/−^ MCs were not statistically significant. In addition, collagen type IV was cleaved after incubation with degranulate from WT MCs, as shown by a decrease in the intensity of the 250 kDa protein band that corresponding to collagen IV (blue-boxed area, Fig. 3C). This effect was significantly reduced when collagen IV was incubated with degranulate from mMCP4^−/−^ MCs, indicating that mMCP4 directly cleaves collagen type IV. Laminin is a trimeric protein with a molecular weight of ~800 kDa that consists of an α-chain (400 kDa), a β-chain (200 kDa) and a γ-chain (200 kDa), which are visible as two protein bands on the blot (Fig. 3D). Laminin was cleaved after incubation with degranulate from both WT and mMCP4^−/−^ MCs (red-boxed area in Fig. 3D indicates cleavage fragments) and no difference was observed between the groups, indicating that mMCP4 does not directly cleave laminin *in vitro* (Fig. 3D).

### Altered gene expression of scar-associated factors in mMCP4 knockout mice after SCI

**Figure 4:**
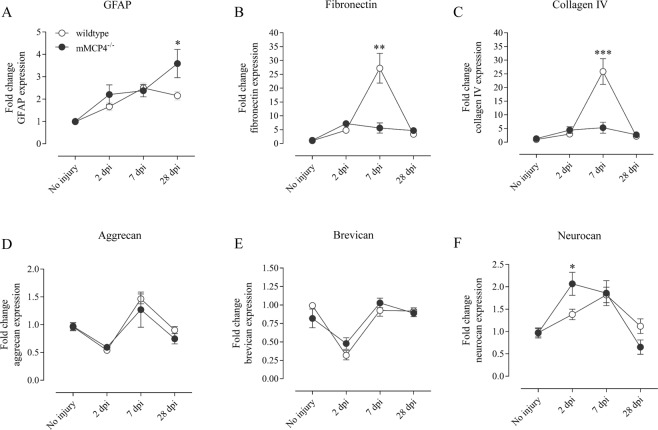
Altered expression of genes coding for scar-associated components after SCI. (**A**–**F**) mRNA expression levels of GFAP, fibronectin, collagen IV, aggrecan, brevican, and neurocan were measured by quantitative PCR analysis in spinal cord tissue from WT (white circles) and mMCP4^−/−^ mice (black circles) at 2 dpi, 7 dpi, and 28 dpi. Samples from mice that did not undergo surgery were included as a control. (**A**) GFAP mRNA levels were significantly elevated in mMCP4^−/−^ mice compared with WT mice at 28 dpi. (**B**,**C**) In contrast, fibronectin and collagen IV gene expression were increased in the WT group compared to mMCP4^−/−^ mice at 7 dpi. Out of the CSPGs, (**D**) aggrecan and (**E**) brevican mRNA were both not differentially expressed after injury whereas the expression of (**F**) neurocan was increased in mMCP4^−/−^ mice compared to the WT mice at 2 dpi. Expression levels were normalized to the reference genes YHWAZ and CYCA, and converted to fold change values vs. the WT control condition using the 2^−∆∆CT^ method as described in the methods. Data are presented as mean ± SEM; n = 5–9 mice analyzed/group; *p < 0.05, **p < 0.01, ***p < 0.001.

Next, we addressed the question of whether endogenous mMCP4 influences the gene expression of important scar-associated markers after SCI. Quantitative PCR analysis showed that GFAP mRNA levels increased slightly after injury in both WT and mMCP4^−/−^ mice (Fig. 4A). At 28 dpi, the expression level was higher in the mMCP4^−/−^ vs. WT mice (fold change in expression vs. WT control condition: 3.590-fold vs. 1.786-fold) (Fig. 4A). For the fibrous ECM component fibronectin, the mRNA levels were decreased at 7 dpi in the mMCP4^−/−^ mice (5.601-fold) compared with WT mice (27.192-fold) (Fig. 4B). Similarly, the expression levels of the basal lamina component collagen IV was decreased 7 dpi in mMCP4^−/−^ mice compared to the WT group (Fig. 4C). We also determined the gene expression profile of selected CSPGs, namely aggrecan, neurocan, and brevican (Fig. 4D–F). The aggrecan mRNA levels decreased at 2 dpi, increased at 7 dpi, after which they decreased again towards baseline levels. No differences were observed between WT and mMCP4^−/−^ mice (Fig. 4D). Similarly to aggrecan, brevican mRNA levels were decreased 2 dpi and no differences were observed between the experimental groups (Fig. 4E). Finally, neurocan mRNA levels were higher in the mMCP4^−/−^ mice (2.069-fold) compared with WT mice (1.382-fold) at 2 dpi (Fig. 4F).

## Discussion

Previously we have shown that MCs play a beneficial role in SCI pathology by reducing scar formation via mMCP6^34^ and by suppressing detrimental inflammatory processes via mMCP4^27,28^. In addition to its immunomodulatory properties, mMCP4 also plays a key role in tissue remodeling and ECM degradation via its own proteolytic capacities or via cleavage-activation of other proteolytic enzymes^16,35^. In this study we show for the first time that mMCP4 targets the inhibitory lesional scar after CNS injury. Similar to our findings in MC-deficient mice, we found that the decline in hind limb motor function in mMCP4 knockout mice (as reported in Nelissen *et al*.^28^) was associated with exacerbated scar formation, *i.e*. elevated expression of axon-growth inhibitory CSPGs in the perilesional area and increased deposition of the fibrotic scar components fibronectin, laminin and collagen IV in the lesion center after SCI. Moreover, we demonstrated that the elevated CSPG levels in the perilesional area and the increased fibrotic scar area were significantly correlated to the impaired motor performance in our mouse model (Figs 1D, I and 2B), highlighting the key role that these factors play in SCI pathology. Several studies have demonstrated that the inhibition of fibrotic scar components and CSPGs promotes regeneration of injured axons and improves functional recovery after CNS injury^36–40^. In a similar way, mMCP4 may degrade ECM components and, thereby, reduce scar tissue formation leading to better motor function. It is tempting to speculate that the larger fibrotic scar in mMCP4 knockout mice is a result of reduced scar compaction due to insufficient degradation of fibronectin, laminin and collagen IV. Increased scar compaction has been positively associated with better functional recovery after SCI^41^. This may in part explain decreased functional recovery in the knockout mice which show reduced scar compaction. Our *in vivo* findings are the first indication that mMCP4 suppresses scarring in the context of CNS trauma. Since scarring has an unfavorable impact on axon regeneration and other repair processes, it is tempting to speculate that the impaired functional outcome in mMCP4 knockout mice may be – at least in part – related to the increased scar formation at the lesion site.

Several studies have revealed that mMCP4 plays a crucial role in tissue remodeling and matrix degradation, both under physiological and pathological conditions^16,33,35^. Chymase/mMCP4 is a chymotrypsin-like protease that has a broad spectrum of activities against various ECM components. For example, it has been shown that mMCP4 has the ability to directly degrade fibronectin, and to indirectly influence ECM remodeling by activating pro-matrix metalloproteinase-2 (pro-MMP2) and pro-MMP9^31,42,43^. In addition, it activates other pro-MMPs such as pro-MMP-1 and pro-MMP-3 that have potent matrix degrading properties^44–46^. Moreover, chymase can inactivate tissue inhibitor of matrix metalloproteinases (TIMPs), thereby increasing MMP activity and matrix remodeling^47^. Our degradation assays, performed with degranulate from WT and mMCP4^−/−^ MCs, showed that collagen IV is a substrate of mMCP4. However, CSPGs and laminin were not cleaved *in vitro*. This finding suggests that additional mechanisms are involved *in vivo* via which mMCP4 reduces scarring after SCI, for example by activating pro-MMPs which in turn cleave ECM components of the fibrotic scars.

In addition, the immunomodulatory effects of mMCP4 may indirectly suppress the scarring response after CNS injury. Previously, we found that mMCP4 displays immunomodulatory functions^28^ by cleaving pro-inflammatory mediators that have detrimental effects after CNS injury. We have demonstrated that IL-6 is upregulated after SCI in mMCP4-deficient mice and is cleaved by mMCP4^28^. Interestingly, IL-6 is a trigger of astrogliosis after SCI^48,49^ and these reactive astrocytes are also the main source of CSPGs produced at the lesion site after injury. Thus, the lack of mMCP4 in our mouse model may increase the deposition of CSPGs via increased IL-6 or other pro-inflammatory cytokines. These findings are in line with increasing evidence suggesting that there is a strong interplay between the immune system and the ECM after CNS injury^50,51^. On the one hand, the glial scar contributes to protection of the spared neural tissues by establishing a boundary between damaged and healthy tissue, and by modulating the immune cells to promote the healing of the CNS tissue^50,51^. On the other hand, reduced fibrotic scar formation can lead to a decreased expression of tumor necrosis factor alpha^52^, which suggests that modulation of the fibrotic scar can also regulate the inflammatory response. Therefore we speculate that mMCP4 may modulate the interplay between the immune response and the scarring response in two ways: mMCP4 may reduce scarring following SCI by modulating inflammatory mediators^27,28^ and it may suppress detrimental inflammatory processes in the injured CNS by cleaving and modifying scar-associated factors^53,54^.

Finally, we investigated whether the absence of mMCP4 affected the expression of selected genes linked to scarring after SCI. We observed increased GFAP mRNA expression levels in mMCP4^−/−^ mice at 28 dpi. Interestingly, the difference in GFAP expression between mMCP4^−/−^ and WT mice in the perilesional area is not reflected at the protein level^28^. In addition, the mRNA levels of the CSPG neurocan were elevated at 2 dpi in the mMCP4^−/−^ mice compared to controls. Surprisingly, in the absence of mMCP4, the gene expression of fibronectin and collagen IV remained low compared to the significantly elevated mRNA levels in WT mice at 7 dpi. In contrast, at the protein level the fibronectin-positive and collagen IV-positive areas were significantly increased in the mMCP4^−/−^ group. It is important to note that the increased levels of fibronectin and collagen IV mRNA are detected early (7 dpi), while the fibronectin and collagen IV protein increases are found at 28 dpi. Moreover, the mRNA data for fibronectin, collagen IV, aggrecan, brevican and neurocan indicate no substantial change at 28 dpi suggesting that the observed differences between mMCP4^−/−^ mice and WT mice on the protein level are the result of degradation, and not of regulatory effects on the gene level or of protein production.

To conclude, in this study, we demonstrate that the absence of mMCP4 in knockout mice results in exacerbated scar formation and this correlated with a reduction in functional recovery after SCI. These data reveal a new mechanism in which endogenous mMCP4 may support recovery after CNS injury via scar remodeling – in addition to its immunomodulatory properties which we have demonstrated previously^28^. Future research will reveal whether therapeutic administration of recombinant mMCP4 improves functional regeneration via scar remodeling and/or modulation of the immune system.

## Methods

### Animals and spinal cord injury

We used mMCP4 knockout mice (mMCP4^−/−^; 10–12 weeks old), which were backcrossed for at least 10-generations to a C57BL/6 background^31^. WT C57BL/6j mice (Janvier) of the same age were used as controls. All mice were housed in a conventional animal facility at Hasselt University under regular conditions, i.e. in a temperature-controlled room (20 ± 3 °C) on a 12 h light-dark schedule and with food and water *ad libitum*. All experiments were approved by the local ethical committee of Hasselt University, and were performed according to the guidelines described in Directive 2010/63/EU.

A T-cut hemisection injury was performed as previously described^34,55^. Briefly, mice were anesthetized by inhalation anesthesia with 3% isoflurane (IsofFlo, Abbot Animal Health) and 0_2_ as a carrier gas; and the spinal cord was exposed by performing a partial laminectomy at thoracic level 8 (T8). Then a bilateral hemisection injury of the spinal cord was induced by using iridectomy scissors to transect the left and right dorsal funiculus, the dorsal horns and the ventral funiculus. Locomotor recovery of the animals at 28 dpi was determined by using the Basso Mouse Scale (BMS)^56^.

### Histological analysis

At 28 dpi, mice received an overdose of Nembutal and they were transcardially perfused with Ringer solution containing heparin, followed by 4% paraformaldehyde in PBS (pH 7.4). Next, 14 µm thick sagittal tissue sections were cut and immunohistochemical stainings were performed as previously described^34^. Spinal cord sections were blocked with 10% normal goat serum and permeabilized with 0.05% Triton X-100 in PBS for 30 min at room temperature (RT). Then, the following primary antibodies were incubated overnight at 4 °C in a humidified chamber: monoclonal mouse anti-glial fibrillary acidic protein (GFAP) (1:500, G3893, Sigma-Aldrich), polyclonal rabbit anti-laminin 1 + 2 (1:200, ab7463, Abcam), polyclonal rabbit anti-fibronectin (1:200, ab2413, Abcam), polyclonal rabbit anti-collagen IV (1:200, ab6586, Abcam) and monoclonal mouse anti-CSPGs (1:200; CS-56; C8035, Sigma-Aldrich). The CS-56 antibody specifically detects the glycosaminoglycan (GAG) portion of native CSPG molecules (e.g. versican, brevican, neurocan). Following repeated washing steps with PBS, spinal cord sections were incubated with Alexa-labeled secondary antibodies for 1 h at RT, namely goat anti-mouse IgG Alexa 555, goat anti-rabbit Alexa 488 and goat anti-mouse IgM Alexa 555 (1:250, secondary antibodies were obtained from Invitrogen). A 4,6-diamino-2-phenylindole (DAPI, Invitrogen) counterstain was performed to reveal cellular nuclei and sections were mounted. Images were taken with a Nikon Eclipse 80i microscope equipped with a Nikon digital sight camera DS-2MBWc. Quantitative image analysis were performed on original unmodified photos using the ImageJ open source software (National Institutes of Health). For standardization, analyses were performed on 5–8 spinal cord sections (per mouse) representing the lesion area (i.e. the lesion epicenter as well as consecutive sagittal sections), as previously described^34,57^. The spinal cord lesion microenvironment can in general be subdivided in different regions, namely the lesion center, the perilesional area and the surrounding tissue. Fibrotic scar tissue is deposited in the lesion center which is surrounded by GFAP-positive astrocytes (=the glial scar)^5,6,38,58^. Therefore, the fibrotic scar area (highlighted in green in Fig. 1Ai/ii) was evaluated by delineating the area in which there was no GFAP-immunoreactivity. To characterize the fibrotic scar in more detail, we also determined the area and immunoreactivity of the following matrix components at the lesion: fibronectin, laminin and collagen IV. To evaluate the expression of CSPGs, intensity analysis of CSPG-immunoreactivity was measured perilesionally in a well-defined area surrounding the lesion center (~200 µm zone surrounding the lesion center). Representative fluorescent photomicrographs are shown in Figs 1A and 2A. To maximise image readability, the contrast and brightness of the stainings was enhanced equally for WT and mMCP4^−/−^ mice.

### Quantitative PCR analysis

mRNA expression levels of glial and fibrotic scar-associated components were investigated at different phases after SCI, namely the acute phase (2 dpi), the subacute phase (7 dpi), and finally the early stage of the chronic remodeling phase (28 dpi). At these selected time points after injury, WT C57BL/6 mice and mMCP4^−/−^ mice were transcardially perfused with Ringer solution as described. Healthy mice (without SCI) were included as controls in the analysis. Standardized areas of spinal cord tissue (5 mm cranial and 5 mm caudal to the lesion center) were collected and mRNA was extracted using the Paris Kit (Life Technologies), according to the manufacturer’s instructions with minor modifications as described in Vangansewinkel *et al*.^34^. Reverse transcription to cDNA (VWR) was performed following the reaction protocol provided with the qScript™ cDNA SuperMix.

Quantitative PCR was conducted on a StepOnePlus detection system (Applied Biosystems) using universal cycling conditions (20 s at 95 °C, 40 cycles of 3 s at 95 °C and 30 s at 60 °C). The reaction mixture contained fast SYBR Green master mix (Applied Biosystems), 10 mM of forward and reverse primers (Eurogentec), RNase free water, and 8 ng template cDNA in a total reaction volume of 10 µl. The primer sequences used are shown in Table 1. Relative quantification of gene expression was accomplished by using the 2^−ΔΔCT^ method and data were normalized to the most stable reference genes. Briefly, GeNorm software identified cyclophilin A (CYCA) and tyrosine 3-monooxygenase/tryptophan 5-monooxygenase activation protein z (YWHAZ) as the most stable reference genes. Allprimers were designed using Primer-Express (http://www.ncbi.nlm.nih.gov/tools/primer-blast). The gene expression levels are presented as fold change of the WT control condition.

**Table 1:** Overview gene-specific primers used for quantitative PCR analysis.

Components	Gene name	Accession number^a^	Primer sequence 5′→ 3′	Amplicon length (bp)
**Gliosis** GFAP	*Gfap*	NM_001131020.1	FW: TCTCCAACCTCCAGATCCGA Rev: CTGGTGAGCCTGTATTGGGA	113
**Scar components** Fibronectin 1	*Fn1*	NM_010233.1	FW: ATGTGGACCCCTCCTGATAGT Rev: GCCCAGTGATTTCAGCAAAGG	124
Collagen, type IV, α I	*Col4a1*	NM_009931.2	FW: AACAACGTCTGCAACTTCGC Rev: CTTCACAAACCGCACACCTG	136
Aggrecan	*Acan*	NM_007424.2	FW: GTCGCTCCCCAACTATCCAG Rev: AAAGTCCAGGGTGTAGCGTG	193
Neurocan	*Ncan*	X84727.1	FW: CACAGAAGTGAGATCAGTGAGA Rev: GCACCATCTTGGTTCAGGCA	114
Brevican	*Bcan*	X87096.1	FW: TGCCGAAGACCTAAATGGAGA Rev: CACGTTCCAGACAGTAGTCCC	89
**Reference genes** CYCA	*Ccna2*	NM_009828.2	FW: GCGTCTCCTTCGAGCTGTT Rev: AAGTCACCACCCTGGCA	108
YWHAZ	*Ywhaz*	NM_011740.3	FW: CAACGATGTACTGTCTCTTTTGG Rev: GTCCACAATTCCTTTCTTGTCATC	149

^a^NCBI accession number of mRNA and corresponding gene, available at http://www.ncbi.nlm.nih.gov/gene GFAP: glial fibrillary acidic protein; CYCA: cyclin A2 YHWAZ: tyrosine 3-monooxygenase/tryptophan 5-monooxygenase activation protein zeta.

### Extracellular matrix degradation assays

Degradation assays were performed to determine which scar-associated ECM components are a substrate of mMCP4 *in vitro*. MC degranulate was obtained from WT and mMCP4^−/−^ mice as previously described^28,34^. Murine recombinant fibronectin (1 µg; Abcam), laminin (0.5 µg; Millipore) or a CSPG-mix (CC117, 2 µg; Millipore) were incubated with 20 µl MilliQ or with 20 µl degranulate obtained from either WT or mMCP4^−/−^ MCs for 48 h at 37 °C. After incubation, samples were mixed with reducing sample buffer and the cleaved fragments were identified via SDS-PAGE and Western blot analysis. Briefly, sample buffer containing 5% β-mercaptoethanol (Fluka Biochemika) was added and the incubation mixture was denaturated at 95 °C for 5 min. Western blotting for collagen IV was performed under non-reducing conditions (no denaturation with β-mercaptoethanol). Protein samples were separated on 7.5% polyacrylamide gels, containing Tris-glycine and transferred onto polyvinylidene fluoride membranes. Membranes were blocked in 5% nonfat powdered milk in tris-buffered saline-Tween 20 (0.1%) (TBST) for 1 h and probed overnight at 4 °C with one of the following primary antibodies: polyclonal rabbit anti-fibronectin (1:1000, sc-9068, Santa Cruz), anti-collagen IV (1:1000, ab6586, Abcam), anti-laminin 1 + 2 (1:1000, ab7463, Abcam) and anti-CSPGs (1:1000, CS56, C8035, Sigma-Aldrich). Next, membranes were washed with TBST and incubated with the appropriate HRP-conjugated secondary antibodies: goat anti-mouse IgM and goat anti-rabbit (dilution 1:5000; all secondary antibodies were obtained from Dako). An ECL Plus detection kit (Thermo Scientific, Pierce®) was used and the generated chemiluminescent signal was detected using a luminescent image analyzer (ImageQuant LAS 4000 mini). Quantitative analysis were performed on original unmodified blots and densitometry of the protein bands (corresponding to the known molecular weight of the respective ECM components – highlighted by blue boxes in the blots of Fig. 3) was quantified via the ImageQuant TL software. For laminin, the density of the two molecular weight bands were summed together (Fig. 3D). The density of the cleavage fragments has not been measured because we cannot guarantee that the used antibodies are able to detect all cleavage fragments because they are produced to target the native ECM protein. Therefore, a reduction in the density of the ECM component protein band suggests a reduction in the amount of protein, or it may indicate cleavage. The densities of the experimental conditions (containing degranulate from WT or mMCP4^−/−^ MCs) have been normalized to the densities of unstimulated control bands. To minimize bias due to differences in densitometric measurements between experiments, each control condition per experiment was set at 100%, thereby lacking a standard error bar. It is important to note that the ECM degradation assays were run in parallel with our previous study^34^ to guarantee comparability between the cleavage effects of mMCP6 and mMCP4 on scar components. This implicates that we used the same control groups for both studies. The control graphs in Fig. 3A–D, i.e. group a (recombinant protein) and group b (recombinant protein + MC_WT_ degranulate) are reprinted with permission from *The Faseb Journal*. To improve readability of the images, the contrast and brightness was modified in the representative protein blots that are displayed in Fig. 3. Original Western blots are shown in Supplementary Fig. S1.

### Statistical analysis

All statistical analyses were performed using GraphPad Prism 5.01 software (GraphPad Software, Inc.). Data sets were analyzed for normal distribution using the D’Agostino-Pearson normality test. Histological differences between WT and knockout mice at 28 dpi were statistically analyzed using the nonparametric Mann-Whitney U test. Individual data points are shown per mouse, and also the corresponding box plots with the median and whiskers indicating the minimum and maximum are presented (Figs 1 and 2). Correlative analysis between the functional outcome and scar formation (fibrotic scar area and CSPG expression) after SCI was performed with the Spearman rank correlation test. *In vitro* ECM degradation assays were analyzed with a one-way ANOVA to compare multiple groups followed by a Tukey *post hoc* test. Quantitative PCR data were analyzed using two-way ANOVA with a Bonferroni *post hoc* test. These data were presented as mean ± standard error of the mean (Figs 3 and 4). At 95% confidence interval, differences were considered statistically significant when p < 0.05.

## Supplementary information

Supplementary materials

## Data Availability

All data supporting the findings of this study are available from the corresponding author on reasonable request.
