# Supplementary material for: Mouse mast cell protease 4 suppresses scar formation after traumatic spinal cord injury
Source: Sci Rep. 2019 Mar 6;9:3715. doi: 10.1038/s41598-019-39551-1 (PMC6403346; doi:10.1038/s41598-019-39551-1)
Supplement: Supplementary file 1 — Supplementary materials [file 41598_2019_39551_MOESM1_ESM.docx]

# Supplementary materials

**Manuscript:**

Mouse mast cell protease 4 suppresses scar formation after traumatic spinal cord injury

**Author names and affiliations:**

Tim Vangansewinkel^1a^, Stefanie Lemmens^1a^, Nathalie Geurts^1^, Kirsten Quanten^1^, Dearbhaile

Dooley^2^, Gunnar Pejler^3,4^ and Sven Hendrix^1*^

^1^Department of Morphology, Biomedical Research Institute, Hasselt University, Diepenbeek, Belgium; ^2^Health Science Centre, School of Medicine, University College Dublin, Dublin, Ireland; ^3^Department of Anatomy, Physiology and Biochemistry, Swedish University of Agricultural Sciences, Uppsala, Sweden and ^4^Department of Medical Biochemistry and Microbiology, Uppsala University, Uppsala, Sweden.

^a^TV and SL contributed equally to this study.

^*^**Corresponding author:**

Sven Hendrix, MD, PhD

Martelarenlaan 42

3500 Hasselt

Belgium

Tel: +32 11 26 92 46; Fax: +32 11 26 92 99

Email: sven.hendrix@uhasselt.be


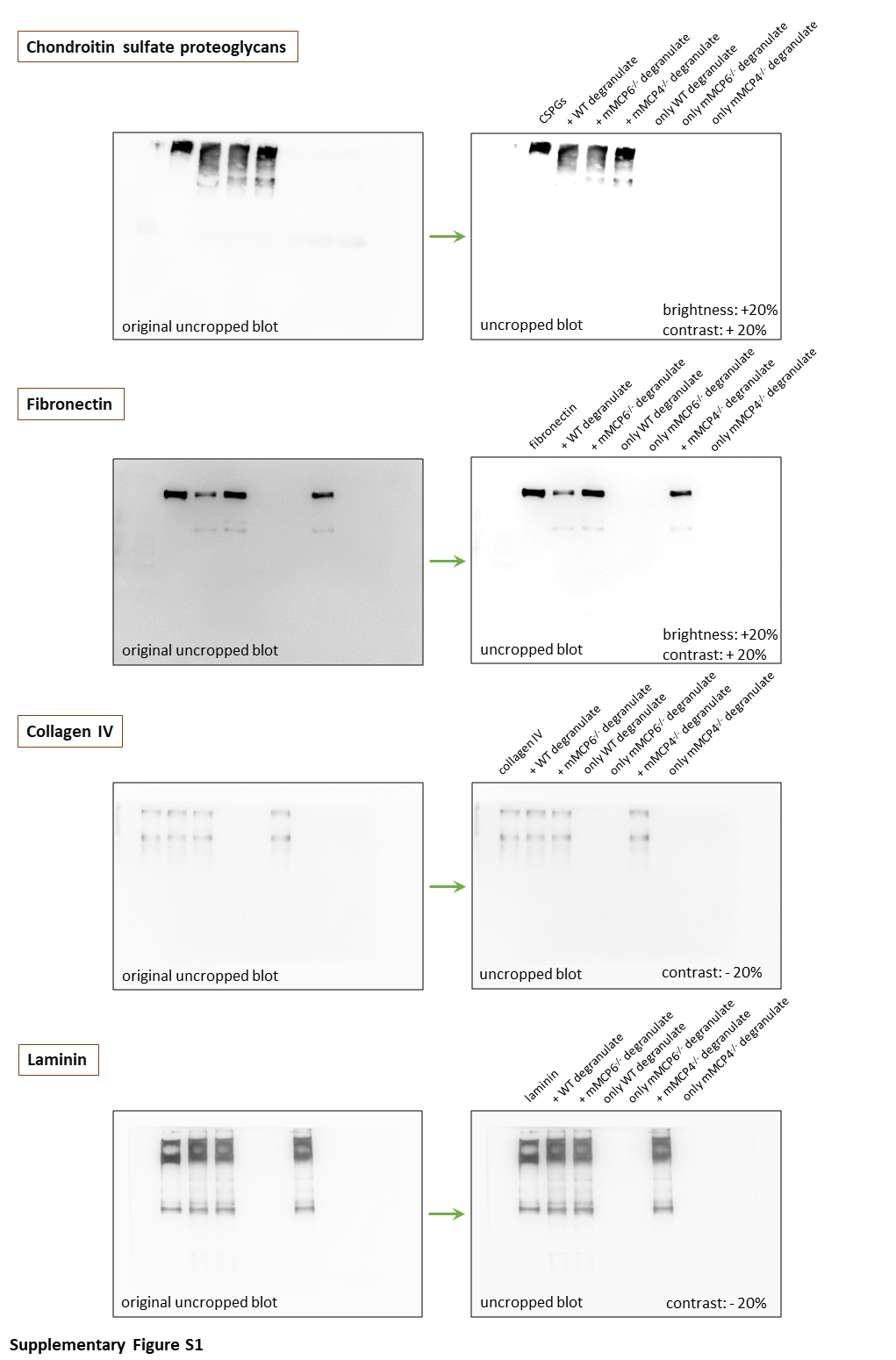


Legend continued on the next page.

**Supplementary Fig. S1 Original unmodified Western blot images of the ECM degradation assay.** The ECM degradation assay shown in Fig. 3A-D was performed in parallel with our previous study on mMCP6 ^34^, in order to guarantee comparability between the cleavage effects of mMCP6 and mMCP4 on scar components. For clarity, we removed the data about mMCP6 from the blots, namely the condition in which mMCP6^‑/-^ degranulate was incubated with and without the respective recombinant ECM protein. Moreover, the contrast and brightness was modified in the representative protein blots to maximize image readability (green arrows indicate that conversion steps were performed).
